# Predicting the Trajectory of Replacements of SARS-CoV-2 Variants Using Relative Reproduction Numbers

**DOI:** 10.3390/v14112556

**Published:** 2022-11-18

**Authors:** Chayada Piantham, Kimihito Ito

**Affiliations:** 1 Graduate School of Infectious Diseases, Hokkaido University, Hokkaido 060-0818, Japan; 2 International Institute for Zoonosis Control, Hokkaido University, Hokkaido 001-0020, Japan

**Keywords:** SARS-CoV-2, relative reproduction number, Delta, Alpha, variant replacement

## Abstract

New variants of the severe acute respiratory syndrome coronavirus 2 (SARS-CoV-2) with high effective reproduction numbers are continuously being selected by natural selection. To establish effective control measures for new variants, it is crucial to know their transmissibility and replacement trajectory in advance. In this paper, we conduct retrospective prediction tests for the variant replacement from Alpha to Delta in England, using the relative reproduction numbers of Delta with respect to Alpha estimated from partial observations. We found that once Delta’s relative frequency reached 0.15, the date when the relative frequency of Delta would reach 0.90 was predicted with maximum absolute prediction errors of three days. This means that the time course of the variant replacement could be accurately predicted from early observations. Together with the estimated relative reproduction number of a new variant with respect to old variants, the predicted replacement timing will be crucial information for planning control strategies against the new variant.

## 1. Introduction

Since its first emergence in the human population in 2019, the severe acute respiratory syndrome coronavirus 2 (SARS-CoV-2) has been generating new variants. Natural selection selects new variants that have higher effective reproduction numbers than other circulating variants. As a result, the average transmissibility in the viral population increases over time [1]. The emergence and replacement among variants of concern (VOCs), Alpha (B.1.1.7), Beta (B.1.351), Gamma (P.1), Delta (B.1.617.2), and Omicron (B.1.1.529) [2] are the process of natural selection.

It is important to know the transmissibility of new variants in comparison with previously circulating variants because the average reproduction number of the circulating virus changes when new variants become dominant. Several studies have analyzed the reproduction numbers of new variants that have replaced old ones. Volz et al., estimated the effective reproduction number of Alpha in England to be 1.5–2.0 times higher than that of non-VOCs using a logistic growth model for relative variant frequencies [3]. Leung et al., estimated the basic reproduction number of Alpha to be 1.75 times higher than that of previously circulating variants in England using a renewal-equation-based model [4]. Ito et al., estimated the effective reproduction number of Delta to be 1.35 times higher than that of Alpha from relative variant frequencies observed in Japan by using an approximated version of the renewal-equation-based model [5]. Using the same method, Ito et al., estimated the effective reproduction number of Omicron to be 3.15 times higher than that of Delta in Denmark [6], and Nishiura et al., estimated the effective reproduction of Omicron to be 4.2 times higher than that of Delta in South Africa [7].

In order to prepare control measures against new variants, it is crucial to predict the trajectory of the variant replacements in advance. The prediction of variant selection has been widely studied in seasonal influenza viruses [8]. Łuksza and Lässig developed a fitness model using mutations on epitopes and non-epitopes to predict selected variants [9]. Huddleston et al., predicted the future relative frequency of variants using its current relative frequency, the antigenic novelty of epitopes, and the mutational load in non-epitopes [10]. Piantham and Ito modeled the fixation probability of variants using relative variant frequency and statistics on patient ages [11]. In the case of seasonal influenza, the main driving force of natural selection was the population immunity acquired from previous infections. In contrast, most of the human population were considered naïve to SARS-CoV-2 at the beginning of the pandemic, and a method to predict the trajectory of variant replacements in the early stage of the pandemic can be simpler than those assuming pre-existing immunity from previous infections.

The transmissibility of an infectious agent can be measured by its reproduction number. The effective reproduction number at time t ($R_{t}$) is defined as the average number of people someone infected at time t could expected to produce if conditions should remain unchanged [12]. When more than one variant of the infectious agent is circulating, the relative reproduction number can be used to measure the relative transmissibility of a variant compared to a baseline variant [4,13]. However, the method requires the numbers of new cases in addition to the relative frequencies of variants, and it is not applicable for predicting variant replacement in the future. Using approximations, Ito et al., proposed a method to determine the relative reproduction number without knowing the number of new cases [5]. This method allows us to predict the future trajectory of variant replacements.

Nucleotide sequences of SARS-CoV-2 variants have been collected worldwide and accumulated in the GISAID database [14]. It is known that different geographical locations have different distributions of variants [15]. As of 28 September 2022, a total of 13,283,666 sequences have been registered on the database worldwide. Of these, 2,286,890 (17.2%) were submitted from England, which has their population account for 0.71% of the world population. These numbers indicate that England is one of the locations with the highest sequencing capacity. In England, the Alpha–Delta replacement was observed from March 2021 to June 2021. The sequence information during the Alpha–Delta replacement in England is one of the best datasets to evaluate the predictability of variant replacement in SARS-CoV-2.

In this study, we conduct retrospective prediction tests using the nucleotide sequences collected in England during the Alpha–Delta replacement. For each given time point, we use partial sequence data observed only up to that time point to estimate the relative reproduction number of Delta with respect to (w.r.t.) Alpha. The estimated relative reproduction number is then used to predict the future trajectory of variant replacement. The estimated relative reproduction numbers and the predicted trajectories are evaluated by being compared to those estimated using the entire dataset.

## 2. Materials and Methods

### 2.1. Nucleotide Sequences

Nucleotide sequences of SARS-CoV-2 viruses collected from England from 1 January 2021 to 31 July 2021 were downloaded from the GISAID database on 16 November 2021. Of these, 411,123 sequences had complete information about the date of sample collection in the metadata. The PANGO lineage names [16] of these sequences were collected from metadata and recorded with their collection dates (Table S1). Sequences that are labeled as “B.1.1.7” or sublineage names starting with “Q.” were classified as the Alpha variant. Sequences that are labeled as “B.1.617.2” or sublineage names starting with “AY.” were classified as the Delta variant. There were 11,773 sequences (2.9%) of lineages other than Alpha and Delta, and these were ignored in subsequent analyses. A total of 399,350 sequences of Alpha (192,250) and Delta (207,100) were used for counting the daily numbers of sequences belonging to Alpha and Delta (Figure S1).

### 2.2. Model of Advantageous Selection

We estimated the relative reproduction number of a variant w.r.t. a baseline variant using an approximated version of the renewal-equation-based model [5]. Let X and Y represent variants circulating in the population and $q_{X}\left(t\right)$ and $q_{Y}\left(t\right)$ denote relative frequencies of X and Y at calendar time t, respectively. Suppose that variant X was dominant at $t_{0}$ and variant Y was introduced into the population at that time with an initial relative frequency of $q_{Y}\left(t_{0}\right)$. We assume that the effective reproduction number of variant Y was k times higher than that of variant X and that k is constant over time. Let $f\left(\tau \right)$ be the probability density function of generation time τ for SARS-CoV-2 infections. We assume $f\left(\tau \right)$ follows the gamma distribution with a shape parameter of 3.42 and a scale parameter of 1.36 [17]. We discretize $f\left(\tau \right)$ to $g\left(j\right)=\int_{j-1}^{j}f\left(\tau \right)d\tau$ for 1≤j≤19. We truncate the generation time distributions at τ>20 and set $g\left(20\right)=\int_{19}^{\infty }f\left(\tau \right)d\tau$ so that $\sum_{j=1}^{20}g\left(j\right)=1$. Let $I\left(t\right)$ be the total number of new infections by either X or Y at calendar time t. Based on Fraser’s time-since-infection model [12], the effective reproduction numbers of variant X and Y can be calculated as

(1) $$R_{X}\left(t\right)=\frac{q_{X}\left(t\right)I\left(t\right)}{\sum_{j=1}^{20}g\left(j\right)q_{X}\left(t-j\right)I\left(t-j\right)}$$

and

(2) $$R_{Y}\left(t\right)=\frac{q_{Y}\left(t\right)I\left(t\right)}{\sum_{j=1}^{20}g\left(j\right)q_{Y}\left(t-j\right)I\left(t-j\right)}.$$

Since the effective reproduction number of variant Y is k times higher than that of variant X, the effective reproduction number of variant Y at time t is given by

*R_Y_*(*t*) = *kR_X_*(*t*). (3)

Assuming that the viral population at time t comprises only variants X and Y, the relative frequency of variant Y at calendar time t, $q_{Y}\left(t\right)$, can be calculated as

(4) $$q_{Y}\left(t\right)=\frac{q_{Y}\left(t\right)I\left(t\right)}{q_{X}\left(t\right)I\left(t\right)+q_{Y}\left(t\right)I\left(t\right)}.$$

We assume that the numbers of new infections do not vary greatly for 20 days, i.e.,

(5) $$I\left(t-1\right)≅\cdots ≅I\left(t-20\right)$$

For $t>t_{0}$. In our previous publication using SARS-CoV-2 data from Denmark, we compared models using Equation (5) with models not using Equation (5). As a result, models using approximation with Equation (5) had lower AIC than their corresponding models without the approximation, suggesting that approximation using Equation (5) gives a better model than that without Equation (5) by eliminating noise in observed $I\left(t\right)$ [18]. Using this approximation with Equations (1)–(3), we can rewrite Equation (4) using $q_{Y}\left(t-j\right)$ for 1≤j≤20 as

(6) $$q_{Y}\left(t\right)=\frac{k\sum_{j=1}^{20}g\left(j\right)q_{Y}\left(t-j\right)}{\sum_{j=1}^{20}g\left(j\right)q_{X}\left(t-j\right)+k\sum_{j=1}^{20}g\left(j\right)q_{Y}\left(t-j\right)}.$$

The average reproduction number of circulating viruses can be determined by the expected value of the reproduction numbers of circulating variants. Since the relative reproduction number of X is 1, and that of Y is k, the average relative reproduction number of circulating viruses at time t w.r.t. variant X is given by

(7) $$q_{X}\left(t\right)+kq_{Y}\left(t\right).$$

### 2.3. Parameter Estimation from the Number of Sequences

Let $N_{X}\left(t\right)$ and $N_{Y}\left(t\right)$ be the number of sequences of variant X and Y observed at calendar time t, respectively. Suppose that variant Y is sampled and sequenced following a beta-binomial distribution having distribution parameters of $\alpha =q_{Y}\left(t\right)M$ and $\beta =\left(1-q_{Y}\left(t\right)\right)M$, where M=α+β. The parameter M represents the sum of the two shape parameters of the underlying beta distribution and it determines how proportions of variants vary during sampling. Note that this beta-binomial distribution has a mean of $\left(N_{X}\left(t\right)+N_{Y}\left(t\right)\right)q_{Y}\left(t\right)$ and a variance of $\frac{\left(N_{X}\left(t\right)+N_{Y}\left(t\right))q_{X}\left(t\right)q_{Y}\left(t\right)(N_{X}\left(t\right)+N_{Y}\left(t\right)+M\right)}{M+1}$. The beta-binomial distribution becomes the binomial distribution when M=∞. To reduce computational time, the upper limit of M is set to 2000. When $q_{Y}\left(t\right)=0.5$ and M=2000, the first and third quartiles of the beta distribution are 0.492 and 0.508, respectively. The following equation gives the likelihood function of parameters k, $q_{Y}\left(t_{0}\right)$, and M for observing $N_{X}\left(t\right)$ and $N_{Y}\left(t\right)$ sequences of variants X and Y at calendar time t:

(8) $$L\left(k,q_{Y}\left(t_{0}\right),M\;;\;N_{X}\left(t\right),N_{Y}\left(t\right)\right)=\left(\begin{array}{c} N_{X}\left(t\right)+N_{Y}\left(t\right)\\ N_{Y}\left(t\right) \end{array}\right)\frac{B\left(N_{Y}\left(t\right)+\alpha ,\;N_{X}\left(t\right)+\beta \right)}{B\left(\alpha ,\beta \right)}$$

where $\alpha =q_{Y}\left(t\right)M$, $\beta =\left(1-q_{Y}\left(t\right)\right)M$, and $B\left(x,y\right)=\frac{\Gamma \left(x\right)\Gamma \left(y\right)}{\Gamma \left(x+y\right)}$. The likelihood for observing $N_{Y}\left(t\right)$ sequences of variant Y during the period on calendar times $t_{1}$, …,$t_{n}$ is given by the product of the above formula for 1≤t≤n.

We consider Alpha and Delta as variants X and Y, respectively. $N_{X}\left(t\right)$ and $N_{Y}\left(t\right)$ are the numbers of sequences of Alpha and Delta in England at calendar time t, respectively. The date of the first introduction of Delta, $t_{0}$, was set to the first date when $N_{y}\left(t\right)>1$ (18 March 2022). The estimates of k, $q_{Y}\left(t_{0}\right)$, M, and $q_{Y}\left(t\right)$ were obtained by maximizing the likelihood function from $t=t_{0}$ until the latest t in which $q_{Y}\left(t\right)<1$ (4 July 2021). We used the Sbplx algorithm in the NLopt library to determine the maximum likelihood [19,20]. The 95% confidence intervals (95% CI) of k, $q_{Y}\left(t_{0}\right)$, and M were determined using the profile likelihood method [21]. Augmented Lagrangian algorithm [22] in the NLopt library was used to determine 95% CIs using Sbplx as the subsidiary optimization algorithm. From the maximum likelihood estimates of k and $q_{Y}\left(t\right)$, the average relative reproduction number of circulating viruses w.r.t. Alpha at time t was estimated from Equation (7). The 95% CIs of $q_{Y}\left(t\right)$ and the average relative reproduction number of circulating viruses w.r.t. Alpha at time t were determined using combinations of parameters within 95% confidence region [21].

### 2.4. Prediction of Relative Variant Frequency and Average Relative Reproduction Number

Relative frequencies of Delta and average relative reproduction numbers of circulating viruses w.r.t. Alpha in the future were predicted using the maximum likelihood estimates of parameters calculated from early observations. For each proportion *p* = 0.05, 0.10, 0.15, 0.20, 0.25, 0.30, 0.35, 0.40, 0.45, 0.50, 0.55, 0.60, 0.65, 0.70, 0.75, 0.80, 0.85, 0.90, and 0.95 we determined the calendar times $s_{p}$ when the estimated relative frequency $q_{Y}\left(s_{p}\right)$ exceeded p using the maximum likelihood estimates calculated with the entire observations from 18 March to 4 July 2021. For each date $s_{p}$ determined above, we calculated the maximum likelihood estimates of k, $q_{Y}\left(t_{0}\right)$, and M using observations no later than $s_{p}$. Relative frequencies of Delta and average relative reproduction numbers of circulating viruses w.r.t. Alpha in the future were predicted by substituting k and $q_{Y}\left(t_{0}\right)$ in Equations (6) and (7), respectively. The 95% CIs of $q_{Y}\left(t\right)$ and the average relative reproduction number of circulating viruses w.r.t. Alpha at time $t>s_{p}$ were determined using combinations of parameters within 95% confidence region estimated from observations at time $t\leq s_{p}$.

## 3. Results

### 3.1. Estimation of Relative Reproduction Number from Entire Observations

Table 1 shows maximum likelihood estimates and their 95% CIs of model parameters calculated from the entire observations in England from 18 March to 4 July 2021. The relative reproduction number (k) of Delta w.r.t. Alpha was estimated to be 1.88 (95% CI: 1.85, 1.91) with a beta-binomial distribution parameter (M) of 288.54 (95% CI: 202.96, 406.26). We call each of these estimates the ‘final estimate’ of each parameter.

**Figure 1:**
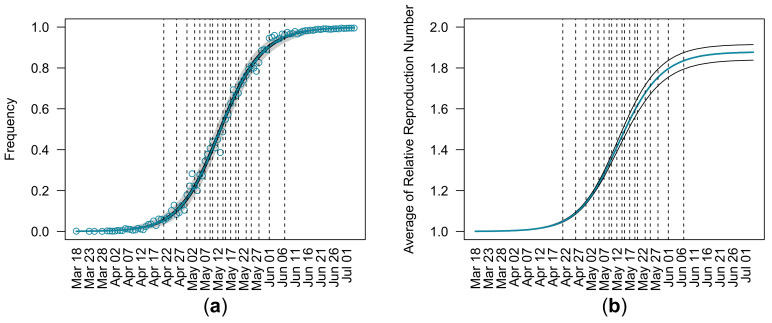
Estimated relative frequencies of the Delta variant and average relative reproduction number of circulating viruses with respect to Alpha using entire observations in England from 18 March to 4 July 2021. (**a**) The observed and estimated relative frequencies of Delta during the Alpha–Delta replacement. Circles represent relative frequencies of Delta sequences collected in England. The blue curve represents the maximum likelihood estimates of relative frequencies of Delta. Black curves surrounding the blue curve represent 95% confidence intervals of the estimated relative frequencies of Delta. Gray area represents the 95% equal-tailed interval of beta distribution for the maximum likelihood estimations of parameters of the estimated beta-binomial distribution. (**b**) The maximum likelihood estimates and 95% confidence intervals of the average relative reproduction number of circulating viruses with respect to Alpha. The blue curve and black curves represent the maximum likelihood estimates and 95% confidence intervals of the average relative reproduction number of circulating viruses with respect to Alpha. Vertical dashed lines in both panels indicate the dates when the estimated relative frequency of Delta reached 0.05, 0.10, 0.15, 0.20, 0.25, 0.30, 0.35, 0.40, 0.45, 0.50, 0.55, 0.60, 0.65, 0.70, 0.75, 0.80, 0.85, 0.90, and 0.95.

Figure 1a shows the observed and estimated relative frequencies of Delta during the Alpha–Delta replacement in England. The blue curve and black curves around the blue curve represent the maximum likelihood estimates and 95% CI of relative frequencies of Delta. The gray area represents 95% equal-tailed intervals of the beta distribution with the parameters $q_{Y}\left(t\right)M$ and $\left(1-q_{Y}\left(t\right)\right)M$. Figure 1b shows the maximum likelihood estimates and 95% CI of the average relative reproduction number of circulating viruses w.r.t. Alpha during the same period. Dashed vertical lines in both panels indicate the dates when relative frequencies of Delta exceeded each 0.05 increment from 0.05 to 0.95 (Table 2). It took 47 days for Delta to reach relative frequencies of 0.05 (21 April 2021) to 0.95 (7 June 2021).

### 3.2. Relative Reproduction Number of Delta with Respect to Alpha Estimated from Partial Data

Table 3 shows the parameters of our model estimated using the partial data collected no later than each of the dates in Table 2. The final estimate of k using observations of the entire period in the Alpha–Delta replacement was 1.88 (Table 1). The final estimate was within 95% CIs of estimations in seventeen out of nineteen estimations using the partial observations. Only the two early estimations, made at relative frequencies of 0.05 and 0.10, failed to cover the final estimate of k in their 95% CIs. All 95% CIs of k estimated at relative frequencies greater than or equal to 0.15 covered the final estimate of k. These results implied that it was possible to accurately estimate the relative reproduction number of Delta w.r.t. Alpha when relative frequencies of Delta became 0.15 or later. It took 38 days for Delta to reach a relative frequency of 0.95 (7 June 2021) from when it was 0.15 (30 April 2021) (Table 2). Therefore, we would be able to estimate the relative reproduction number of Delta w.r.t. Alpha more than one month before its fixation.

### 3.3. Prediction of Relative Variant Frequency in Future

**Figure 2:**
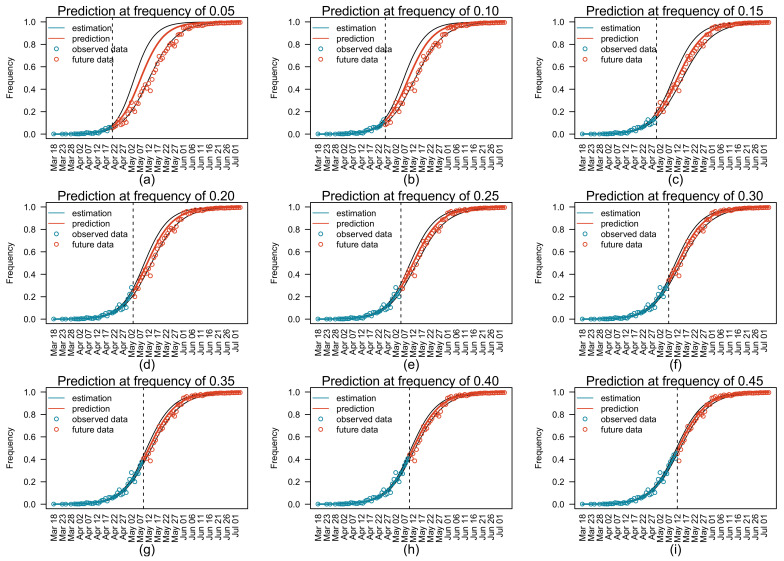
Prediction of future relative frequencies of the Delta variant using partial observations. Panels (**a**–**i**) represent predictions estimated using observations until 21 April, 26 April, 30 April, 3 May, 5 May, 7 May, 9 May, 10 May, and 12 May 2021, respectively. Blue circles represent observed relative frequencies used for predictions. Red circles represent future observations that were not used for predictions. The vertical dashed line in each panel represents the date of the last observations used for prediction. The blue curve in each panel represents the maximum likelihood estimates of relative frequencies of Delta, and the red curve represents the relative frequencies predicted by the model using the estimated parameters. Black curves represent 95% confidence intervals of the relative frequencies of Delta.

We conducted retrospective prediction tests on the future relative frequency of Delta and the average relative reproduction number of circulating viruses w.r.t. Alpha using model parameters in Table 3, which were estimated from partial observations. Figure 2 shows predicted trajectories of the Alpha–Delta replacement using partial observations up to different time points in Table 2. The maximum likelihood predictions made at relative frequencies of 0.05 and 0.10 overestimated the future relative frequencies of Delta (Figure 2a,b), while predictions made at relative frequencies greater than or equal to 0.15 fitted well with future observations (Figure 2c–i).

**Figure 3:**
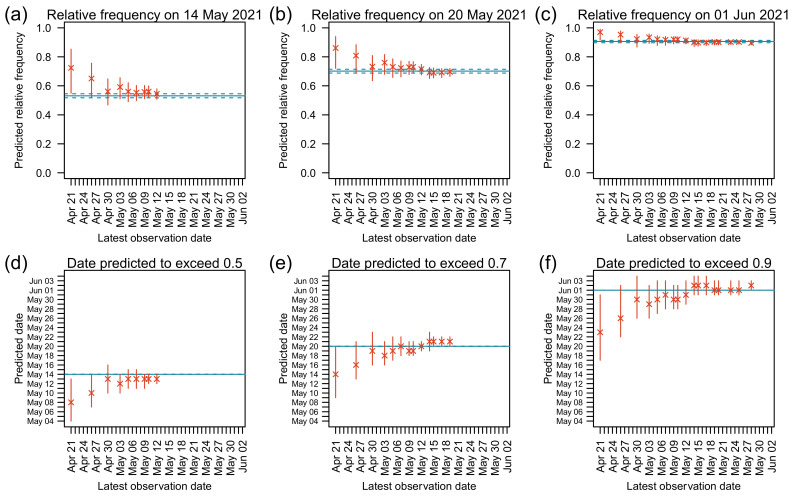
Predictions of relative frequencies of Delta on target dates (**a**–**c**), and predictions of the dates when Delta would reach target relative frequencies (**d**–**f**). In each panel, x-axis represents dates until which observations were used in the prediction. Y-axes in panels (**a**–**c**) represent the predicted relative frequencies on 14 May, 20 May, and 1 June, respectively. Y-axes in panels (**d**–**f**) represent the predicted dates when Delta would reach relative frequencies of 0.50, 0.70, and 0.90, respectively. Cross marks represent predicted relative frequencies and dates with vertical bars showing their 95% confidence intervals. The blue horizontal solid lines represent the final estimates using the entire observations. The blue horizontal dashed lines represent 95% confidence intervals of the final estimates.

According to the final estimate using the entire observations, Delta exceeded relative frequencies of 0.50, 0.70, and 0.90 on 14 May, 20 May, and 1 June 2021, respectively (Table 2). We evaluated the accuracy of predictions by analyzing predictions targeted on these dates (Figure 3). When predictions were made before the relative frequencies of Delta reached 0.15, the relative frequencies of Delta on the target dates were overestimated (Figure 3a–c) and the dates predicted to exceed target relative frequencies were earlier than the final estimates (Figure 3d–f). The reason for these was that these early predictions overestimated the relative reproduction numbers of Delta w.r.t. Alpha (Table 3). In contrast, predictions made when relative frequencies of Delta were greater than or equal to 0.15 were close to the final estimate of relative frequencies (Figure 3a–c) and dates (Figure 3d–f). When relative frequencies of Delta were greater than or equal to 0.15, the predicted relative frequencies targeted on 14 May, 20 May, and 1 June 2021 had median errors of 0.060 (*n* = 7), 0.023 (*n* = 11), and 0.004 (*n* = 15) with maximum absolute errors of 0.092 (*n* = 7), 0.060 (*n* = 11), and 0.034 (*n* = 15), respectively (Table 4). With the same setting, the predicted dates exceeding targeted relative frequencies of 0.50, 0.70, and 0.90 had median errors of 1 (*n* = 7), 1 (*n* = 11), and 1 (*n* = 15) days with maximum absolute errors of 2 (*n* = 7), 2 (*n* = 11), 3 (*n* = 15) days, respectively (Table 5).

## 4. Discussion

We analyzed the replacement from the Alpha variant to the Delta variant in England using nucleotide sequences on the GISAID database collected from 18 March to 4 July 2021. The estimated relative reproduction number, k, of Delta w.r.t. Alpha was 1.88 (95% CI: 1.85–1.91) with a beta-binomial distribution parameter (M) of 288.54 (95% CI: 202.96–406.26) (Table 1). The relative reproduction number of Delta w.r.t. Alpha was accurately estimated from early observations once the relative frequencies of Delta reached 0.15 (Table 3). Using these estimates of the relative reproduction number, the date when the relative frequency of Delta would reach 0.90 was predicted with a maximum absolute prediction error of three days (Table 5).

Several studies have estimated the relative reproduction of Delta w.r.t. Alpha in different countries. Ito et al., estimated the relative reproduction number of Delta w.r.t. Alpha in Japan to be 1.35 [5]. Hansen estimated the relative reproduction number of Delta w.r.t. Alpha in Denmark to be 2.17 [23]. In this study, the relative reproduction number of Delta w.r.t. Alpha was estimated to be 1.88 (95% CI: 1.85–1.91) (Table 1). Figgins and Bedford found that the relative reproduction number of Delta and Alpha w.r.t. non-VOC variants in the United States were different depending on the states [24]. The differences in relative reproduction numbers of Delta w.r.t. Alpha among countries or states may be attributed to the differences in vaccine usage or the ethnicity of the target populations.

Our model assumes that the sequences on the GISAID database were sampled following a beta-binomial distribution. We can use the binomial distribution in the model instead of the beta-binomial distribution. The model using beta-binomial distribution resulted in lower Akaike information criteria (AIC) compared to the model using binomial distribution (Table S2). This means that the observed variance was larger than the variance of the binomial distribution. The additional variance to the binomial distribution may be attributed to the difference between relative variant frequencies among subpopulations, indicating that the target population was not well-mixed. For example, different regions may show different progresses in the variant replacement. The same may be true for different age groups.

It is possible to use a logistic regression model to fit the trajectory of the relative frequency of variants to achieve the same purpose as in this study. The renewal-equation-based model described in this study resulted in a lower AIC than using a multinomial logistic regression model [25] as shown in Table S3. This suggested that the renewal-equation-based model is a better model than the logistic regression-based model for this dataset.

The lockdown restrictions in the UK were relaxed on 17 May 2021. The relative reproduction number estimated using data up to 28 May 2021 was slightly lower than the final estimation using entire observations (Table 3). This is attributed to the decrease in the relative frequency of Delta and the increase of the relative frequency of Alpha around 28 May 2021 (Figure 1). The underlying mechanism of the drop in the relative frequency of Delta after the relaxation of lockdown is unknown and needs to be further investigated.

Prediction tests conducted in this study used the date of sample collection and did not consider the delay in sequence submissions. Nucleotide sequences collected during the period from 18 March 2021, the introduction of Delta, to 13 May 2021, the date when Delta reached a relative frequency of 0.95, were submitted after 10.78 days on average with a standard deviation of 3.76 days. Indeed, 95% of sequences during this period were submitted to the GISAID database within 16 days after sample collection. This means real-time prediction may need additional 10–16 days to achieve a prediction accuracy similar to the results shown in this study.

Our model assumes that there was no difference between the generation times of both variants with a mean value of 4.64 days [17]. However, Hart et al., estimated the generation time of Delta (4.7 days) to be shorter than that of Alpha (5.5 days) [26]. To allow differences between generation times of variants, it is necessary to extend the model to also estimate the relative generation times of the variant w.r.t. that of the baseline variant [18].

During the period of analysis, the percentage of people receiving two-dose vaccinations in England was increasing from 7% on 1 April to 39% on 1 June 2021 [27]. Our model assumes that there was no difference between vaccination efficacy against Alpha and Delta. If there was a difference between vaccine efficacies against Alpha and Delta, the relative reproduction number of Delta w.r.t Alpha would change as population vaccine coverage increases. Detection of the difference in vaccine efficacy among variants may be possible by analyzing the temporal change of relative reproduction number using vaccination coverage data if there is a sufficient difference.

## 5. Conclusions

In this study, we showed that the relative reproduction number of Delta w.r.t. Alpha in England and its future trajectory of replacement could be predicted one month before it reached a relative frequency of 0.90. Public health policy-makers would have had only one month to prepare control measures for the increase in viral transmissibility. This implies that a quick decision-making process is needed to take advantage of the prediction. This period can be extended if accurate predictions are available using data earlier than one month. Further research is needed to investigate how much additional information was required for obtaining accurate predictions using data earlier than one month.

## Figures and Tables

**Table 1:** Parameters estimated using the entire observations during the Alpha–Delta replacement in England.

k (95% CI)	$q_{Y}\left(t_{0}\right)$ (95% CI)	M (95% CI)	Log Likelihood
1.88 (1.85, 1.91)	0.0005 (0.0004, 0.0006)	288.54 (202.96, 406.26)	−431.00

**Table 2:** Maximum likelihood estimates for dates (yyyy-mm-dd) when Delta exceeded certain relative frequencies and the average relative reproduction numbers w.r.t. Alpha on those dates.

Relative Frequency	Date When Delta Exceeded the Relative Frequency (95% CI)	Average Relative Reproduction Number w.r.t. Alpha (95% CI)
0.05	2021-04-21 (2021-04-16, 2021-04-24)	1.049 (1.045, 1.053)
0.10	2021-04-26 (2021-04-23, 2021-04-29)	1.090 (1.084, 1.095)
0.15	2021-04-30 (2021-04-27, 2021-05-02)	1.141 (1.134, 1.148)
0.20	2021-05-03 (2021-04-30, 2021-05-05)	1.193 (1.185, 1.203)
0.25	2021-05-05 (2021-05-03, 2021-05-07)	1.235 (1.223, 1.246)
0.30	2021-05-07 (2021-05-05, 2021-05-09)	1.281 (1.267, 1.295)
0.35	2021-05-09 (2021-05-07, 2021-05-10)	1.332 (1.314, 1.349)
0.40	2021-05-10 (2021-05-08, 2021-05-12)	1.358 (1.339, 1.377)
0.45	2021-05-12 (2021-05-10, 2021-05-14)	1.412 (1.389, 1.434)
0.50	2021-05-14 (2021-05-12, 2021-05-15)	1.467 (1.440, 1.493)
0.55	2021-05-15 (2021-05-13, 2021-05-17)	1.493 (1.465, 1.521)
0.60	2021-05-17 (2021-05-15, 2021-05-19)	1.545 (1.513, 1.577)
0.65	2021-05-19 (2021-05-17, 2021-05-21)	1.594 (1.559, 1.628)
0.70	2021-05-20 (2021-05-18, 2021-05-23)	1.617 (1.580, 1.652)
0.75	2021-05-23 (2021-05-20, 2021-05-25)	1.677 (1.638, 1.716)
0.80	2021-05-25 (2021-05-23, 2021-05-28)	1.712 (1.671, 1.751)
0.85	2021-05-28 (2021-05-25, 2021-05-31)	1.754 (1.713, 1.794)
0.90	2021-06-01 (2021-05-29, 2021-06-05)	1.796 (1.754, 1.837)
0.95	2021-06-07 (2021-06-03, 2021-06-13)	1.835 (1.794, 1.875)

**Table 3:** Parameters estimated using partial observations.

Observed Relative Frequency	k (95% CI)	$q_{Y}\left(t_{0}\right)$ (95% CI)	M (95% CI)	Log Likelihood
0.05	2.15 (2.00, 2.45)	0.0002 (0.0001, 0.0003)	834.12 (346.81, 2000.00 ^†^)	−80.91
0.10	2.06 (1.92, 2.21)	0.0002 (0.0001, 0.0004)	581.40 (268.62, 1426.19)	−102.87
0.15	1.93 (1.83, 2.05)	0.0004 (0.0002, 0.0006)	399.87 (202.44, 832.82)	−123.23
0.20	1.97 (1.87, 2.08)	0.0003 (0.0002, 0.0005)	362.81 (188.91, 720.53)	−137.69
0.25	1.92 (1.83, 2.02)	0.0004 (0.0002, 0.0006)	307.07 (165.69, 575.00)	−148.88
0.30	1.91 (1.83, 2.00)	0.0004 (0.0003, 0.0006)	310.63 (170.09, 574.06)	−158.03
0.35	1.92 (1.85, 2.00)	0.0004 (0.0003, 0.0006)	328.39 (182.12, 607.05)	−166.02
0.40	1.93 (1.86, 2.00)	0.0004 (0.0003, 0.0006)	339.98 (189.18, 628.24)	−170.45
0.45	1.90 (1.84, 1.96)	0.0004 (0.0003, 0.0006)	315.00 (179.08, 565.97)	−180.84
0.50	1.86 (1.79, 1.92)	0.0005 (0.0004, 0.0008)	231.61 (136.25, 392.22)	−194.90
0.55	1.85 (1.79, 1.91)	0.0006 (0.0004, 0.0008)	234.52 (139.84, 401.81)	−198.95
0.60	1.86 (1.80, 1.91)	0.0005 (0.0004, 0.0007)	247.76 (147.88, 419.60)	−207.59
0.65	1.87 (1.81, 1.92)	0.0005 (0.0004, 0.0007)	248.77 (150.01, 415.23)	−217.85
0.70	1.86 (1.81, 1.91)	0.0005 (0.0004, 0.0007)	250.70 (152.17, 417.80)	−222.51
0.75	1.86 (1.82, 1.91)	0.0005 (0.0004, 0.0007)	271.02 (164.92, 448.18)	−235.19
0.80	1.87 (1.82, 1.91)	0.0005 (0.0004, 0.0007)	285.77 (174.67, 473.56)	−244.46
0.85	1.84 (1.81, 1.89)	0.0006 (0.0004, 0.0007)	250.12 (159.20, 426.80)	−261.43
0.90	1.86 (1.82, 1.90)	0.0005 (0.0004, 0.0007)	238.70 (154.56, 365.00)	−284.10
0.95	1.88 (1.85, 1.92)	0.0005 (0.0004, 0.0006)	209.79 (142.17, 313.90)	−321.85

^†^ The upper bound of M in the maximum likelihood estimation was set to 2000.

**Table 4:** Errors of predicted relative frequencies on target dates.

Target Date	Final Estimate of Relative Frequency	Number of Predictions ^†^	Absolute Errors in Predicted Relative Frequency	
			Median	Maximum
14 May 2021	0.50	7	0.060	0.092
20 May 2021	0.70	11	0.023	0.060
1 June 2021	0.90	15	0.004	0.034

^†^ The two earliest predictions, made when Delta was less than 0.15, were excluded.

**Table 5:** Errors of predicted dates exceeding target relative frequencies.

Target Relative Frequency	Final Estimate of Date	Number of Predictions ^†^	Absolute Errors of Predicted Dates	
			Median	Maximum
0.50	14 May 2021	7	1	2
0.70	20 May 2021	11	1	2
0.90	1 June 2021	15	1	3

^†^ The two earliest predictions, made when Delta was less than 0.15, were excluded.

## Data Availability

Table S1 contains metadata of all nucleotide sequences of SARS-CoV-2 viruses collected from England from 1 January 2021 to 31 July 2021 used for the analysis.

## Supplementary Materials

The following supporting information can be downloaded at: https://www.mdpi.com/article/10.3390/v14112556/s1, Figure S1: Daily relative variant frequencies of Alpha (red), Delta (blue), and other variants (gray) in England from 1 January 2021 to 31 July 2021 calculated from nucleotide sequences on the GISAID database; Table S1: Metadata of nucleotide sequences of SARS-CoV-2 viruses collected from England during 1 January 2021 to 31 July 2021; Table S2: Parameters estimated by the binomial distribution model and comparison of AIC values with those of the beta-binomial distribution model; Table S3: Comparison between AIC values of the renewal-equation-based model and that of the logistic regression model estimated from entire observation.
