# Supplementary material for: Predicting the Trajectory of Replacements of SARS-CoV-2 Variants Using Relative Reproduction Numbers
Source: Viruses. 2022 Nov 18;14(11):2556. doi: 10.3390/v14112556 (PMC9697243; doi:10.3390/v14112556)
Supplement: Supplementary file 1 [file viruses-14-02556-s001.zip › viruses-1973123-supplementary.pdf]

viruses

MDPI

Supplementary Materials

Predicting the Trajectory of Replacements of SARS-CoV-2 Variants Using Relative Reproduction Numbers

Chayada Piantham [1] and Kimihito Ito [2,*]

[1] Graduate School of Infectious Diseases, Hokkaido University
[2] International Institute for Zoonosis Control, Hokkaido University
* Correspondence: itok@czc.hokudai.ac.jp

Supplementary Materials

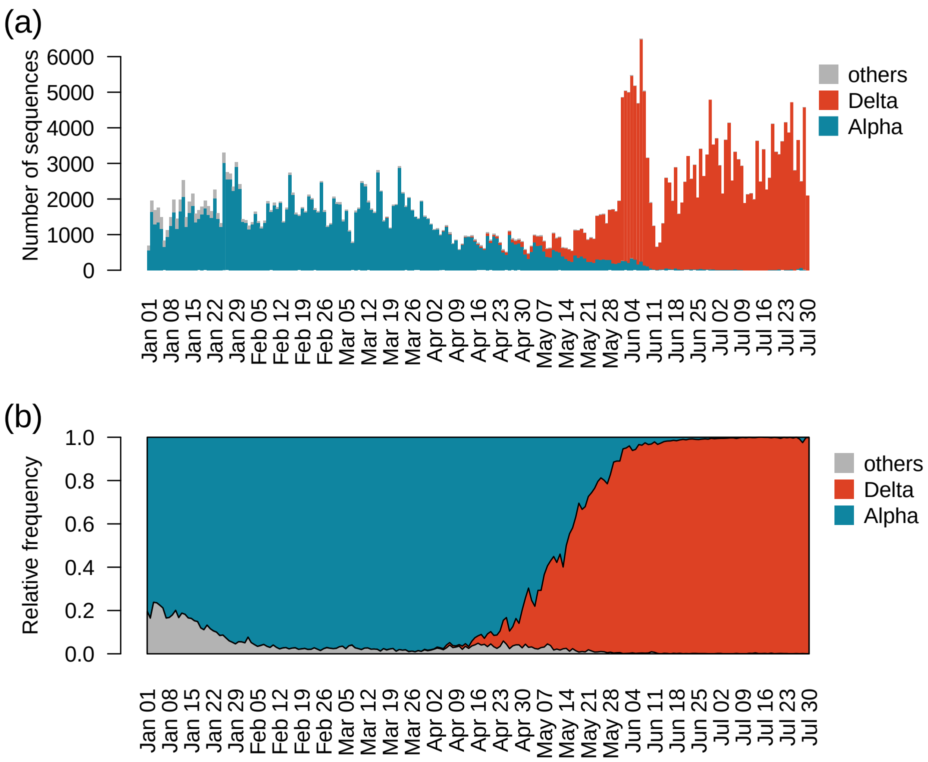

Figure S1. Daily variant frequencies of Alpha (red), Delta (blue), and other variants (gray) in England during 1st January 2021 to 31st July 2021 calculated from nucleotide sequences on the GISAID database.

Table S1. Metadata of nucleotide sequences of SARS-CoV-2 viruses collected from England during 1st January 2021 to 31st July 2021. (please see the excel file)

Table S2. Parameters estimated from entire observation by the binomial distribution model and comparison of AIC values with that of the beta-binomial distribution model.

Model	k (95% CI)	$q_Y(t_Y)$ (95% CI)	M (95% CI)	Log likelihood	AIC[†]
Beta-binomial distribution	1.88 (1.85, 1.91)	0.0005 (0.0004, 0.0006)	288.54 (202.96, 406.26)	–431.00	868.00
Binomial distribution	1.92 (1.91, 1.93)	0.0003 (0.0003, 0.0004)	–	–643.06	1292.12

[†]AIC refers to the Akaike information criterion of the model.

Table S3. Comparison between AIC values of the renewal-equation-based model and that of the logistic regression model estimated from entire observation.

Model	k (95% CI)	$q_Y(t_Y)$ (95% CI)	Intercept (95% CI)	M (95% CI)	Log likelihood	AIC
Renewal-equation-based	1.88 (1.85, 1.91)	0.0005 (0.0004, 0.0006)	–	288.54 (202.96, 406.26)	–431.00	868.00
Logistic regression	1.93 (1.92, 1.97)	–	0.12 (0.12, 0.13)	235.67 (186.14, 393.88)	–435.24	876.48
